# OP55 Defining And Classifying Digital Health Technologies: A Review Of Literature And Delphi Consensus Approach

**DOI:** 10.1017/S026646232510113X

**Published:** 2025-12-29

**Authors:** Annapoorna Prakash, Marcia Tummers, Wietske Kievit, Liza van Mun, Jeremy Diez, Zoe Garrett, Sonia Muñoz-López, Joan Segur-Ferrer, Livio Battaglia, Michela Santurri, Simona Paone, Wija Oortwijn

## Abstract

**Introduction:**

To support decision-making regarding the introduction of digital health technologies (DHTs) with added value for patients and society, health technology assessment (HTA) frameworks for DHTs are needed. However, the absence of standardized definitions and taxonomies creates a barrier to the development of such a framework. This research addressed these challenges by developing a consensus-based unified definition and taxonomy for DHTs.

**Methods:**

The study comprised a scoping review of existing definitions of DHTs, a targeted review of taxonomies used across European countries, and a two-round Delphi process. For the scoping review, data on the source identifier, type of DHT, the definition itself, and the purpose of the definition were extracted. For identified taxonomies, data on criteria of classification (e.g., functionality, risk, user), types of DHTs, and background of the classification (development process, purpose, and policy context) were extracted. Thematic analysis of both reviews informed the development of an online Delphi survey, distributed via several HTA stakeholder groups and societies, including HTAi.

**Results:**

We identified 8,651 unique records from four databases (PubMed, the Cochrane Library, Embase, EconLit) and extracted 328 definitions. Analysis revealed three key components of a definition for DHT: intended purpose, users, and underlying technology. The targeted review identified nine taxonomies classifying DHTs by maturity stages, intended purpose, digital health architecture, illness severity, personalization capacity, and levels of human intervention. The first round of the Delphi survey, with 118 complete responses, achieved consensus on the three key components, using intended purpose as a classification criterion. The results of the second round will be available in January 2025.

**Conclusions:**

This study provided a unified definition for DHTs, incorporating three key components: intended purpose, users, and underlying technology. The Delphi process validated intended purpose as a primary classification criterion. This standardized definition and taxonomy streamlines assessment, enhances cross-border HTA collaboration, and enables stakeholders to compare and evaluate the impact of DHTs appropriately, thus fostering informed decision-making.

